# Exercise-Driven Increase in Gut Microbial Hydrogen Production as a Possible Factor of Metabolic Health

**DOI:** 10.3389/fphys.2020.01065

**Published:** 2020-09-02

**Authors:** Sergej M. Ostojic

**Affiliations:** FSPE Applied Bioenergetics Lab, University of Novi Sad, Novi Sad, Serbia

**Keywords:** exercise, gut microbiota, molecular hydrogen, metabolism, signaling molecule

Barton et al. (2018) have recently evaluated the differences in fecal microbiota between professional rugby players and healthy age-matched sedentary controls. The authors reported a rather preferential intestinal microbiome profile in physically active individuals, characterized by the greater microbial diversity and functional/metabolic activity, as evaluated by whole metagenome shotgun sequencing. It appears that increased amino acids and carbohydrates metabolism, along with microbiota-driven short-chain fatty acids (SCFAs) upturn, were associated with enhanced fitness (coupled with dietary adjustments) and overall health when compared to inactive counterparts. In spite of many unresolved issues, an affirmative link between exercise-diet and metabolic health could be attributed to the compositional structure of the gut microbiota and its metabolites. I agree with this solid conclusion and present here a credible hypothesis that the beneficial metabolic effects might also involve an exercise-driven increase in microbial production of hydrogen gas (H_2_).

H_2_ has recently been conceded as a biologically active gas that functions as a selective antioxidant, anti-inflammatory and anti-apoptotic agent, and signaling molecule (Ohta, 2014). An abundant metabolite of gut flora, hydrogen acts as a preventive and therapeutic gas in diverse animal models and human diseases, while a possible imbalance in its homeostasis may adversely affect human health. For instance, a deficient output of endogenous H_2_ has been implicated in several gut microbiota-mediated diseases (Ostojic, 2018; Smith et al., 2019), whereas a surplus in bacteria-generated H_2_ after a dietary intervention appeared to be associated with better cardiometabolic health (Suzuki et al., 2009). A recent trial has indicated that exercise by itself may have similar effects, and upregulate hydrogen-specific bacteria to instigate positive metabolic adaptations. Allen et al. (2018) reported that hydrogen-producing bacterial genera (e.g., *Clostridiales* spp., *Lachnospira* spp., *Roseburia* spp.) significantly increased in abundance after 6-week exercise training in previously sedentary lean and obese women, and decreased after a return to sedentary activity. Exercise-induced modulations of the gut microbiota found in this study were strongly associated with favorable changes in several biomarkers of metabolic fitness (e.g., body composition, VO_2max_), suggesting a possible interconnection between exercise stimulus, compositional and functional changes in the gut microbiota that involve hydrogen, and better metabolic health. An earlier study demonstrated that acute exercise augments breath H_2_ excretion after lactulose test (Ehrenpreis et al., 2002), an outcome corroborated in a recent gut-exercise challenge study (Gaskell et al., 2020), implying the colonic bacteria as a source of extra endogenous hydrogen during exercise.

An exercise-driven production of additional H_2_ likely appears due to the higher activity of hydrogen-releasing bacteria, but it might be accompanied by a decreased (or relatively inferior) activity/abundance of hydrogenotrophs (e.g., sulfate-reducing bacteria, methanogens, acetogens); a net gain of each component to gut hydrogen turnover remains unaddressed. Theoretically, an increased fermentation due to exercise-driven gut hypoxia along with higher utilization of lactates as a prevailing metabolic substrate during exercise may contribute to favoring hydrogen-producing microbiota (Allen et al., 2018), with extra H_2_ perhaps available to manifest its metabolic footprint in the human body (Suzuki et al., 2009; Ohta, 2014). A discrete effect of this hydrogen on specific biomarkers of metabolic health is currently unknown but may be partly responsible for the overall gut-derived functional-metabolic benefits of the exercise demonstrated in a previous study (Barton et al., 2018). Monitoring the luminal production of H_2_ and its pharmacokinetics-pharmacodynamics profiles after exercise for systemic metabolic sequels are thus highly warranted to back up this hypothesis, while accounting for individual variation in microbiota profiles, exercise regimens (e.g., single session vs. long-term exercise) and other environmental stimuli, in well-sampled robust longitudinal RCTs.

In this article, I principally discussed a possibility that the beneficial metabolic effects of exercise could involve an exercise-driven increase in gut microbial hydrogen production. However, other metabolites of gut microbiota (e.g., SCFAs, sulfates) could also step in the process, while endogenous hydrogen could be even used up as a substrate by hydrogenotrophic bacteria (Smith et al., 2019). This requires a careful monitoring of gut hydrogen during exercise and perhaps consider its balance in line with individual microbiota profiles that could encompass distincive abundances of hydrogen-producing and hydrogen-consuming genera. To further stipulate microbiota-driven metabolic benefits of exercise, one should also account for other dietary factors, such as a diet rich in fiber that allows gut bacteria to produces more SCFAs (Barton et al., 2018). In addition, exercise-induced alterations of the gut microbiota appear dependent on obesity status (Allen et al., 2018), with exercise increased fecal metabolites in lean, but not obese, participants. These findings implies that the degree of obesity and leanness has a contributory effect on gut microbiota, and this was seen also in subjects who had undergone bariatric surgery (Palmisano et al., 2020). In addition, an increased production of hydrogen gas by gut bacteria could also have adverse effects. For instance, additional hydrogen may hypothetically induce bloating and belching that might compromise normal peristalsis and intestinal epithelium function, or imperil bacterial species that can modulate metabolic use of lactates from exercise (Huang et al., 2019), which requires further investigation.

## Author Contributions

The author confirms being the sole contributor of this work and has approved it for publication.

## Conflict of Interest

The author declares that the research was conducted in the absence of any commercial or financial relationships that could be construed as a potential conflict of interest. The handling Editor declared a past co-authorship with the author SO.
